# Promising Insecticidal Efficiency of Essential Oils Isolated from Four Cultivated *Eucalyptus* Species in Iran against the Lesser Grain Borer, *Rhyzopertha dominica* (F.)

**DOI:** 10.3390/insects13060517

**Published:** 2022-05-31

**Authors:** Asgar Ebadollahi, Bahram Naseri, Zahra Abedi, William N. Setzer, Tanasak Changbunjong

**Affiliations:** 1 Department of Plant Sciences, Moghan College of Agriculture and Natural Resources, University of Mohaghegh Ardabili, Ardabil 5697194781, Iran; 2 Department of Plant Protection, Faculty of Agriculture and Natural Resources, University of Mohaghegh Ardabili, Ardabil 5697194781, Iran; bnaseri@uma.ac.ir (B.N.); zahra.abedi@uma.ac.ir (Z.A.); 3 Aromatic Plant Research Center, 230 N 1200 E, Suite 100, Lehi, UT 84043, USA; setzerw@uah.edu; 4 Department of Pre-Clinic and Applied Animal Science, Faculty of Veterinary Science, Mahidol University, Nakhon Pathom 73170, Thailand

**Keywords:** essential oil, *Eucalyptus*, toxicity, biochemical disruption, nutritional indices, *Rhyzopertha dominica*

## Abstract

**Simple Summary:**

*Eucalyptus* essential oils have shown promising insecticidal effects on several insect pests. The lesser grain borer, *Rhyzopertha dominica* (F.), causes economically significant damage to stored grains as an internal primary insect pest. In this study, the chemical compositions of essential oils isolated from four *Eucalyptus* species. *E. microtheca*, *E. procera*, *E. spatulata*, and *E. torquata* were detected and identified using a gas chromatography-mass spectrometer, and their lethal and sublethal insecticidal effects were evaluated against the adults of *R. dominica*. Although all essential oils have significant fumigant toxicity, due to the high relative potency, *R. dominica* was more susceptible to the *E. procera* essential oil than the others. The total protein, glycogen, and lipid contents and digestive amylase and protease enzyme activities of the treated insects were reduced. The nutritional indices consumption index, relative consumption rate, and relative growth rate were also reduced in the treated adults. The findings of this study reveal that *E. microtheca*, *E. procera*, *E. spatulata*, and *E. torquata* essential oils can be potentially used for the development of eco-friendly natural agents for the management of *R. dominica*.

**Abstract:**

The lesser grain borer, *Rhyzopertha dominica* (F.), causes damage to stored grains resulting in both quantitative and qualitative losses. The use of synthetic fumigants in the management of stored-product pests resulted in undesirable side effects such as environmental contamination and threat to human and animal health. In this study, the lethal and sublethal effects of essential oils from four *Eucalyptus* species, *E. microtheca*, *E. procera*, *E. spatulata*, and *E. torquata* were studied against *R. dominica* adults. Gas chromatographic–mass spectral analysis of the essential oils was carried out, in which terpenes such as 1,8-cineole and globulol were abundant in essential oils. The pest was susceptible to the fumigation of the essential oils and, considering concentrations and exposure times (24, 48, and 72 h), had significant effects on the pest mortality. The total protein, glycogen, and lipid contents and digestive amylolytic and proteolytic activities of the adults treated with tested essential oils were reduced. The consumption index, relative consumption rate, and relative growth rate were also reduced in the treated adults. According to the insecticidal effects on the adults of *R. dominica*, the essential oils of *E. microtheca*, *E. procera*, *E. spatulata*, and *E. torquata* can be candidates for further investigations as grain protectant agents.

## 1. Introduction

The lesser grain borer, *Rhyzopertha dominica* (F.) (Coleoptera: Bostrichidae), an internal primary insect pest of stored products, has a high capacity for locating on grain foods including wheat, barley, maize, rice, and sorghum [1,2]. The insect readily infests storage grains and can cause economic losses throughout much of the world due to its high potential viability and adaptability [3,4,5]. Although the use of chemical fumigants is the main strategy in the management of *R. dominica*, their negative side effects, including environmental contamination and toxicity to human and animal health, are cautioned [6,7]. Furthermore, the resistance of *R. dominica* to phosphine [8,9] and some currently used insecticides [10,11] was reported in recent years. Therefore, the introduction of novel, efficient, and eco-friendly agents should be a priority in pest management research.

The insecticidal potential of essential oils isolated from different aromatic plant families, such as Apiaceae, Asteraceae, Lamiaceae, Myrtaceae, Rutaceae, Verbenaceae, and Zingiberaceae, was reported against coleopteran insect pests [12,13,14]. The susceptibility of *R. dominica* to some plant-derived essential oils was also demonstrated in recent studies. Along with noteworthy acute toxicity, fumigation with *Gaultheria procumbens* L. essential oil presented high anti-nutritional effects and biochemical disturbances against the adults of *R. dominica*, which was attributed to the presence of its major component methyl salicylate [15]. In the study of Ncibi et al. [16], the significant concentration-dependent fumigant toxicity of *Mentha pulegium* L. and *Lavandula stoechas* L. essential oils was reported against *R. dominica*, in which essential oils were rich in terpenic compounds, including pulegone, *iso*-menthone, camphor, and 1,8-cineole. In another study, the essential oil of *Citrus aurantium* L. enriched with terpenes limonene and β-myrcene was characterized by its high fumigant toxicity against the adults of *R. dominica* [17].

*Eucalyptus* (Myrtales: Myrtaceae) is one of the widely-distributed genera in tropical and subtropical regions due to its ease of cultivation, rapid growth, and high adaptability [18]. The essential oil is considered to be the main product of *Eucalyptus* trees, which are widely used in the food and perfume industries and medicine [19]. Terpenes such as 1,8-cineole, cryptone, *p*-cymene, α-pinene, α-terpineol, limonene, and spathulenol are commonly found as the main components of *Eucalyptus* essential oils [20,21,22]. The insecticidal properties of essential oils isolated from several *Eucalyptus* species were demonstrated against coleopteran insect pests, even on *R. dominica*, which was mainly dependent on the presence of terpene compounds [23]. Hamdi et al. [24] reported that the susceptibility of *R. dominica* to the essential oil of *E. lehmani* (Schauer) Benth was greater than those of *Callosobruchus maculatus* F. (Chrysomelidae) and *Tribolium castaneum* Herbst (Tenebrionidae). They concluded that the toxicity of *E. lehmani* essential oil could be ascribed to its chemical composition, mainly 1,8-cineole (34.6%). In another study, along with concentration- and time-dependent fumigant toxicity, the essential oil of *Eucalyptus floribunda* Huegel had significant anti-nutritional effects on the adults of *R. dominica* [25]. In a study by Filomeno et al. [26], among 12 *Eucalyptus* essential oils, the essential oil of *E. resinifera* Sm., due to the presence of a high amount of 1,8-cineole, was identified to show more efficient fumigant activity against the adults of *R. dominica*.

Therefore, to introduce eco-friendly and effective agents, the present study investigates the insecticidal efficacy of essential oils isolated from four *Eucalyptus* species grown in Iran, *E. microtheca* Muell, *E. procera* Dehnh, *E. spatulata* Hook, and *E. torquata* Luehm, against *R. dominica* adults. Along with lethal fumigant toxicity, their anti-nutritional effects and biochemical effects on the exposed adults, including energy resources content and esterase and amylase and protease enzyme activities, were also assessed. In addition, the chemical profile of essential oils was also explored to discuss the possible relationship of components with studied insecticidal potential.

## 2. Materials and Methods

### 2.1. Plant Materials and Extraction of Essential Oils

Leaves of four cultivated *Eucalyptus* species in Iran, *E. microtheca*, *E. procera*, *E. spatulata*, and *E. torquata*, were gathered from the Kashan botanical garden (33°59′20″ N, 51°28′38″ E), Kashan, Iran. The average rainfall, annual temperature, and relative humidity are 139 mm, 19.1 °C, and 20%, respectively, in the region. The trees were usually irrigated at one-week intervals in low rainfall and soil drying conditions. The average age of the trees was about 10 years, and they were not chemically treated in the last two years. Voucher samples were located at the herbarium institute according to their scientific names. The leaves of each species were air-dried at room temperature and after about 10 days, pulverized with an electric grinder. The extraction of essential oils was performed using a Clevenger apparatus with a 2000-mL flask, 1200 mL distilled water, and 100 g of ground leaves within 3 h. Na_2_SO_4_ was used to remove the water from extracted essential oils, which were separately transferred to glass containers, covered by aluminum foil, and stored in a refrigerator at 4 °C.

### 2.2. Chemical Profile of the Essential Oils

Chemical compositions of the *Eucalyptus* essential oils were analyzed by GC-MS using an Agilent 7890B series Gas Chromatography (GC) combined with Agilent 5977A Series Mass Spectrometer (MS) (Santa Clara, CA, USA). The MS was operated in the EI mode (electron energy = 70 eV), scan range = 10–550 amu, and scan rate = 3.99 scans/s. The GC column was an HP-5ms fused silica capillary column with the following features: 30 m length, 0.25 mm diameter, and 0.25 μm film thickness. The carrier gas was helium with a column head pressure of 53.1 kPa and a flow rate of 1.0 mL/min. Inlet temperature was 280 °C, and interface temperature was 280 °C. The GC oven temperature program was used as follows: 50 °C initial temperature, hold for 1 min; increased at 8 °C/min to 100 °C; increased at 6 °C/min to 110 °C, hold for 1 min; then at 6 °C/min to 310 °C, hold for 1 min. A 1% *w*/*v* solution of each essential oil sample in methanol as solvent was prepared, and 1 μL was injected under splitless mode. The essential oil components were tentatively identified by comparing mass spectral fragmentation patterns and retention indices (RI) based on a series of homologous C_8_–C_20_ *n*-alkanes with those reported in databases [27,28,29].

### 2.3. Insect Rearing

The initial population of *R. dominica* was prepared as a colony by the Department of Plant Protection, University of Mohaghegh Ardabili, Iran. The pest was reared in cylindrical plastic containers with openings covered by a mesh cloth for ventilation. After pouring 200 g of crushed wheat (Aftab cultivar), the adult insects were released into the containers and kept in a growth chamber (28 ± 1 °C temperature, 60 ± 5% relative humidity, and 14:10 h light:dark). Adults were removed from the rearing containers after 48 h, and the seeds infected with the pest eggs were stored in the growth chamber under the conditions described. One-day-old adult insects were used for the experiments. Adults that did not move with the stimulation of a hot needle were considered dead [30]. 

### 2.4. Fumigant Toxicity

To evaluate the fumigant toxicity of *Eucalyptus* essential oils, 20 one-day-old adult beetles were transferred to 140-mL glass containers as fumigant chambers. The calculated concentrations of essential oils (14.28, 19.78, 27.92, 39.42, 55.69, and 78.54 µL/L of air for *E. microtheca*; 10.71, 15.92, 23.21, 33.84, 48.91, 71.40 µL/L of air *E. procera*; 21.42, 28.27, 37.85, 50.98, 68.83, and 92.82 µL/L of air for *E. spatulata*, and 17.85, 24.63, 33.27, 44.84, 60.48, 82.11 µL/L of air for *E. torquata*) were poured onto filter paper discs (3 cm in diameter) by micropipette. The range of concentrations, which resulted in about 25 to 75% mortality of the treated insects in the preliminary experiments, was selected, and the other concentrations were determined based on logarithmic intervals. The treated filter papers were glued to the lid of the glass containers using adhesive tape. To prevent evaporation of the essential oils, the lid of each glass container was sealed with a parafilm. Each experiment was repeated three times, and in the control groups, all steps, except adding essential oil, were performed. Mortality of treated insects was recorded after 24, 48, and 72 h intervals. The lids of the fumigant containers were closed immediately after removing the insects and counting their mortality after 24 and 48 h exposure times. 

### 2.5. Biochemical Assays

Calculated 24 h LC_30_ (lethal concentration to kill 30% of treated insets) values were selected for sublethal bioassays: 12.60 µL/L of air for *E. microtheca*, 10.66 µL/L of air for *E. procera*, 21.84 µL/L of air for *E. spatulata*, and 19.01 µL/L of air for *E. torquata*. All reagents and enzyme substrates were purchased from Sigma Chemical Co. (St. Louis, MI, USA). Fast Blue RR salt and bovine serum albumin were obtained from Merck Co. (Darmstadt, Germany) and Roche Co. (Penzberg, Germany), respectively. All assays were repeated three times.

#### 2.5.1. Energy Reserves

The whole bodies of 100 alive adults (one day old) treated with LC_30_ of essential oils within 24 h were homogenized using a hand-held glass homogenizer at 4 °C. The resulting homogeneous mixtures were centrifuged at 10,000 rpm for 10 min (Sigma 1–14 K refrigerated centrifuge, USA). The method described by Van Handel [31] was used to measure the lipid content of adult insects using a vanillin reagent. The Bradford method, using bovine serum albumin as a standard, was used to estimate the protein content [32]. To measure the glycogen content, the anthrone reagent was used, and its absorption was recorded spectrophotometrically at 626 nm (Unico, UV/Vis 2100, Fairfield, NJ, USA) [33].

#### 2.5.2. Esterase Activity

Fifty treated adult insects were individually homogenized with 250 μL of 0.04 M sodium phosphate buffer, pH 7.0, on the ice and centrifuged at 10,000 rpm for 15 min. The supernatant was separated as an enzymatic extract and stored at −20 °C. To measure the activity of α- and β-esterases, 12.5 μL of the enzyme extract was mixed with 112.5 μL of sodium phosphate buffer and 50 μL of α-naphthyl acetate (α-NA; for α-esterase) and β-naphthyl acetate (β-NA; for β-esterase), incubated at 30 °C for 15 min. Then, 50 mg of Fast Blue RR salt in 50 mL of sodium phosphate buffer (0.075 M, pH 7.0) was added. The absorbance was recorded for α-NA and β-NA during 7 min intervals at 450 and 540 nm, respectively, using a microplate reader (ELIZA-Reader, Anthos 2020, Cambridge, UK) [34].

#### 2.5.3. Amylolytic and Proteolytic Activity

To measure the proteolytic activity, azocasein substrate based on the method of Elpidina et al. [35] was used. In total, 80 μL of 1.5% azocasein solution in 2-morpholinoethanesulfonic acid buffer (MES: 50 mM, pH 6.0) was mixed with 20 μL enzyme and incubated at 37 °C for 50 min. The enzymatic reaction was blocked by adding 100 μL of 30% trichloroacetic acid (TCA). The unhydrolyzed azocasein was precipitated by refrigeration at 4 °C for 0.5 h and then centrifuged at 15,000 rpm for 10 min. One hundred μL of sodium hydroxide (2 M) was added to 100 μL of the supernatant, and the absorbance was recorded at 440 nm. In the blank, the enzyme extract was added to the reaction mixture after adding 30% TCA. Each of the experiments related to treatments and control was performed in three replications. The protease activity unit was defined as a change in optical density per milligram of protein per minute.

To measure the amylolytic activity, each experimental unit consisted of 500 μL of acetate buffer (50 mM, pH 6.0), 20 μL of enzyme extract, and 40 μL of 1% soluble starch. Thirty minutes after the reaction at 37 °C, 100 microliters of dinitrosalicylic acid reagent (DNSA: Sigma Chemical Co., St. Louis, MI, USA) were added and then boiled in a water bath for 10 min. After centrifugation at 15,000 rpm at 4 °C for 5 min, the absorbance was recorded at 540 nm using a spectrophotometer. Three replications were considered for each treatment and blank. The amylase activity unit was defined as the quantity of enzyme required to produce 1 mg of maltose at 37 °C [36].

### 2.6. Nutritional Indices of Insect Pest

Two hundred one-day-old adults were treated with LC_30_ of essential oils for 24 h. The surviving insects were divided into 7 groups (10 insects for each) and transferred into 6 cm Petri dishes containing 3 g of crushed wheat seeds (Aftab cultivar). The adults’ weight, food consumption, and weight gain of *R. dominica* were determined after two weeks. The fed and unfed adults and the initial and remaining food were weighed after two weeks (Sartorius AG Germany GCA803S, d = 0.001 ct). To determine the percentage of dry weight of *R. dominica* and food, 20 samples were weighed, dried in an oven (60 °C for 48 h), and re-weighed. The recorded data were used to calculate nutritional indices, including the consumption index (CI), relative consumption rate (RCR), relative growth rate (RGR), and efficiency of conversion of ingested food (ECI), through the following formulae [37]:

Consumption Index = F/A Relative Consumption Rate = F/TA Relative Growth Rate = G/TA Efficiency of Conversion of Ingested food = G/F

where F = dry weight of food eaten (mg), T = feeding period (day), A = mean dry weight of insect during feeding period (mg), and G = dry weight gain of insect during feeding period (mg).

### 2.7. Statistical Analysis

The normality of the data was checked by Kolmogorov–Smirnov test [38]. The lethal concentrations (LC), lethal times (LT), 95% confidence limits, concentration–mortality regression line details, and an χ^2^ test to evaluate data heterogeneity, were also performed. All data were subjected to one-way analyses of variance (ANOVA), and means were separated with the least significant difference (LSD) test. Statistical software SPSS version 16.0 (IBM, Chicago, IL, USA) was used for all statistical analyses.

## 3. Results

### 3.1. Chemical Profile of Essential Oils

Gas chromatographic–mass spectral analysis of the *Eucalyptus* essential oils was carried out (Table 1). 1,8-Cineole dominated all four of the *Eucalyptus* essential oils in concentrations of 12.1%, 21.3%, 27.1%, and 24.2% for *E. microtheca*, *E. procera*, *E. spathulata*, and *E. torquata*, respectively. Likewise, globulol was abundant in all four essential oils (5.0%, 7.2%, 11.3%, and 8.4%, respectively). The additional major components in *E. microtheca* were α-pinene (5.6%), β-pinene (5.1%), and aromadendrene (11.7%), while α-pinene (14.0%), *trans*-pinocarveol (5.0%), *trans*-sabinol (14.6%), and aromadendrene (6.1%) were abundant constituents of *E. procera* essential oil. *trans*-Sabinol (7.6%) was also abundant in *E. spathulata*. *Eucalyptus torquata* essential oil showed high concentrations of α-pinene (20.0%) and aromadendrene (7.8%).

### 3.2. Fumigant Toxicity

Based on the Kolmogorov–Smirnov test, data on the mortality of *R. dominica* adults affected by the essential oils of *E. microtheca*, *E. procera*, *E. spatulata*, and *E. torquata* had significantly normal distributions. According to the results of ANOVA, concentrations of essential oils and exposure times (24, 48, and 72 h) had significant effects on pest mortality. However, interactions between concentration and exposure time had no significant effects on pest mortality except for *E. procera* essential oil (Table 2).

The LC_50_ (Lethal Concentration to kill 50% of the pest population) of *E. microtheca* essential oil was 25.261 µL/L of air after 24 h, which significantly decreased to 18.995 µL/L of air by increasing exposure time to 72 h. A decreasing LC_50_ value with increasing exposure time was also observed for all other essential oils. On the other hand, the susceptibility of *R. dominica* to *Eucalyptus* essential oils was time-dependent. Additionally, due to the low 72 h-LC_50_ and high relative potency, *R. dominica* adults were more susceptible to the *E. procera* essential oil than others. According to LC_90_ values, 37.778 µL/L of air of *E. procera* essential oil will be sufficient for 90% mortality of the pest within 72 h (Table 3). Based on the high *r*^2^ values presented in Table 3, there is a positive and direct correlation between the concentrations of essential oils and insect pest mortality.

### 3.3. Energy Reserves

The effects of the sub-lethal concentration (LC_30_) of *Eucalyptus* essential oils on energy reserves, including the protein, glycogen, and lipid content, of *R. dominica* adults are shown in Table 4. The essential oils significantly decreased the protein and glycogen contents of the pest as compared with the control (*p* < 0.05). The essential oil from *E. procera* led to the highest reduction in protein content. There was a significant reduction in the lipid content of adults treated with *E. procera* and *E. microtheca* essential oils (Table 4).

### 3.4. Esterase Activity

According to Table 5, the esterase activity of adults treated with LC_30_ of *Eucalyptus* essential oils was augmented. The α-esterase activity in the adults treated with *E. microtheca*, *E. procera*, and *E. spatulata* essential oils was more than in the adults treated with *E. torquata* oil and the control (*p* < 0.05). The β-esterase activity in the adults treated with *E. procera*, *E. spatulata*, and *E. torquata* essential oils was more than in the control (Table 5).

### 3.5. Amylolytic and Proteolytic Activity

Enzymatic disruption effects of the LC_30_ of *Eucalyptus* essential oils on amylolytic and proteolytic activities of *R. dominica* adults are presented in Table 6. Essential oils extracted from all *Eucalyptus* species significantly reduced the amylolytic and proteolytic activities of treated insects (*p* < 0.05). Although essential oils of *E. microtheca* and *E. procera* had the highest effects on amylolytic and proteolytic activities, there were no significant differences between their bio-effects with other essential oils (Table 6).

### 3.6. Nutritional Indices

Anti-nutritional effects of *Eucalyptus* essential oils on LC_30_-treated adults of *R. dominica* are presented in Table 7. The consumption index and relative consumption rate were significantly reduced in the adults treated with *E. microtheca*, *E. procera*, and *E. spatulata* essential oils (*p* < 0.05). Treatment with essential oils caused significant decreases in the relative growth rate of adult insects (*p* < 0.05). There were no significant differences in the efficiency of conversion of ingested food between treated and untreated adults (Table 7).

## 4. Discussion

Terpenes such as 1,8-cineole, α-pinene, and aromadendrene are generally abundant constituents of *Eucalyptus* essential oils [39,40]. In this study, the analysis of the *Eucalyptus* essential oils by gas chromatography–mass spectrometry revealed that the terpene compounds 1,8-cineole and globulol were abundant in all *Eucalyptus* essential oils. Furthermore, α-pinene and aromadendrene were among the other major compounds of the essential oils. Previous accounts of the leaf essential oil compositions of *E. microtheca* cultivated in Iran were reported: The essential oil of *E. microtheca* from Kashan province showed 1,8-cineole (34.0%), *p*-cymene (12.4%), α-pinene (10.7%), and β-pinene (10.5%) as the main components [41], which were identified with a different percentage in the present study (12.1%, 0.9%, 5.6%, and 5.1, respectively). *Eucalyptus microtheca* essential oil from Sistan and Baluchestan province, was rich in α-phellandrene (16.5%), aromadendrene (12.8%), α-pinene (6.8%), globulol (6.0%), ledene (5.7%), *p*-cymene (5.3%), and β-pinene (5.0%) [42]. In contrast, the leaf essential oil of *E. microtheca* from central areas of Iran was dominated by γ-gurjunene (22.0%), *p*-cymen-7-ol (10.7%), and aromadendrene (10.5%) [43]. α-Phellandrene and *p*-cymen-7-ol were not recognized in this study, and clearly, there is much variation in *E. microtheca* essential oils. The essential oil compositions of *E. procera* from Iran were investigated in recent studies [44,45]. The identified compounds are comparable to the findings in this study, in which 1,8-cineole (35.9% and 45.0%) and α-pinene (25.6% and 28.6%), respectively, were also dominant. A notable difference, however, was the large concentration of *trans*-sabinol (14.6%) in the present study, which was not indicated in the previous reports. Previous investigations of *E. spathulata* essential oil from Iran have shown 1,8-cineole (72.5% and 67.8%) and α-pinene (12.7% and 8.4%), respectively, as the main components [41,43], in agreement with the present study. However, there are some differences in the identified compounds. For example, *trans*-sabinol, which had a high percentage (7.6%) in the *E. spathulata* essential oil investigated in this study, was not identified in the above-mentioned works [41,43]. The leaf essential oil of *E. torquata*, cultivated in Kashan, Iran, was dominated by 1,8-cineole (66.9%) along with α-pinene (13.9%), *trans*-pinocarveol (6.3%), and globulol (1.6%) [41], which had completely different percentages in the present study: 24.2%, 20.0%, 0.2%, and 8.4%. Another investigation of the leaf essential oil of cultivated *E. torquata* from Kashan, Iran, revealed 1,8-cineole (28.6%), α-pinene (15.7%), and globulol (13.1%) to be the major components [46], while Nikbakht et al. reported 1,8-cineole (69.6%), α-pinene (9.5%), *allo*-aromadendrene (7.8%), and aromadendrene (4.5%) as the major constituents [43]. In this study, *allo*-aromadendrene was identified with a much lower percentage (1.8%) in *E. torquata* essential oil, while the quantity of aromadendrene (7.8%) was higher than that of Nikbakht et al.’s study [43]. Accordingly, there are notable quantitative and qualitative differences in essential oil compositions with *Eucalyptus* species. The differences can be caused by several factors, including environmental conditions and stresses, genetic make-up, geographical origin, phonological stages of plants, distillation time and drying methods, and agricultural practices, affecting the essential oil profiles [47]. 

The insecticidal effects of essential oils can be attributed to their main compounds [15,48]. Additionally, the synergism between other minor constituents can also affect the bioactivities of essential oils [49,50,51]. The fumigant toxicity of borneol, 1,8-cineole, and thymol, identified with different quantities in all *Eucalyptus* essential oils examined in this study, was reported against the adults of *R. dominica* [52]. The fumigant toxicity and antifeedant effect of limonene, as one of the terpenes identified in *E. microtheca* essential oil, was documented against *R. dominica* adults [53]. In the study of Liu et al., the essential oil of *Artemisia nakaii* Pamp and its abundant terpenes camphor and 1,8-cineole showed pronounced fumigant activity against the third instar larvae of *Spodoptera litura* Fab [54]. They found that camphor and 1,8-cineole had better fumigant toxicity than the essential oil and concluded that these terpenes might be the substances responsible for essential oil toxicity. In addition, the anti-nutritional and acetylcholinesterase inhibitory effects of β-Selinene, another terpene identified in this essential oil and the essential oils investigated in the present study, were reported. In another investigation on *Eucalyptus* essential oils and their compounds, the prominent fumigant toxicity of *E. resinifera* essential oil and 1,8-cienole was reported against *R. dominica* [26]. Furthermore, *p*-cymene and α-pinene alone had no fumigant toxicity on *R. dominica*, but in combination with 1,8-cineole, they were more effective than the commercial insecticide pirimiphos-methyl [26]. Therefore, it can be said that the insecticidal properties of *Eucalyptus* essential oils studied in the present study are probably related to their compounds, and dominant terpenes such as 1,8-cineole and even synergism between other compounds, including thymol, limonene, *p*-cymene, and α-pinene, are involved in the observed biological effects.

In the present study, along with the acute fumigant toxicity of *Eucalyptus* essential oils, sublethal chronic effects, including a reduction in protein, glycogen, and lipid contents, inhibition of digestive amylolytic and proteolytic activities, and anti-nutritional effects, on treated insects were also observed. Since proteins, carbohydrates and lipids play vital roles in the physiological pathways of insects, from growth, reproduction and metamorphosis to diapause, and the resistance to low temperature, reducing the content of these macromolecules could be considered an important mechanism in their control [55,56,57]. In this study, even though decreases in lipid content by *E. spatulata* and *E. torquata* essential oils treatments were not significant (*p* < 0.05), the energy content of *R. dominica* adults treated with all *Eucalyptus* essential oils was reduced in terms of total protein, glycogen, and lipids amount. According to the present findings, the amylolytic and proteolytic activity was also decreased in *R. dominica* adults treated with all *Eucalyptus* essential oils compared with controls. A decrease in such digestive enzyme activities by the plant-derived essential oils was previously reported [58,59,60]. Similar to our findings, the essential oil of *E. globulus* Labill was able to reduce the amylase and protease activity of *Ephestia kuehniella* Zeller larvae (Lepidoptera: Pyralidae) [59]. It was suggested that the digestive enzyme synthesis could be reduced by plant secondary metabolites, including essential oils and their constituents, through cytotoxic impact and structural alteration of gut epithelial cells and decreasing metabolism rate [60,61]. The nutritional indices, including consumption index (CI), relative consumption rate (RCR), relative growth rate (RGR), and efficiency of conversion of ingested food (ECI), were diminished in the adults of *R. dominica* treated with all *Eucalyptus* essential oils. However, the decrease in ECI was not significant in comparison with the control group. Similarly, RCR, RGR, and ECI in the adults of *R. dominica* were decreased by essential oils of three other *Eucalyptus* species: *E. dundasii* Maiden, *Eucalyptus floribundi* Hugel ex Endi, and *E. kruseana* Muel [25,62,63]. The insects’ growth and consumption rate were dependent on the food quality. When food is not good quality, the insect avoids eating it or eats less [64]. Furthermore, if the food consumed by the insect is not absorbed, the insect’s body size and weight will be reduced [64]. In this study, *Eucalyptus* essential oils showed a great insecticidal potential on the adults of *R. dominica*, in which digestive amylase and protease enzymes, nutritional indices, and subsequent energy reserves were decreased.

Essential oils have developed as secondary metabolites in plants against herbivores, and plant-herbivore coevolution continues [65]. Faced with complex mixtures of constituents in essential oils, diverse mechanisms correspondingly evolve in insect pests to overcome such secondary metabolites [66]. An increase in the levels of detoxifying enzymes is one of the key mechanisms for developing insect resistance to insecticidal agents [67]. Among detoxifying enzymes, esterases deserve more attention because they can be involved in metabolizing various exogenous and endogenous compounds [68]. Although the β-esterase activity of *R. dominica* treated with *E. microtheca* was not significantly different from the control in our study, over-production of α- and β-esterases was detected in the adults treated with *Eucalyptus* essential oils (*p* < 0.05). It can be concluded that the pest may show a degree of resistance to *Eucalyptus* essential oils due to the increase in esterase enzymes. However, it should also be noted that the essential oils have multiple modes of action, such as the inhibition of acetylcholinesterase and glutathione *S*-transferases and disruption in the octopamine receptors [12,14]. However, the inhibitory effects of *Eucalyptus* essential oils on the digestive enzymes profile and the reduction of energy reserves of the pest are also demonstrated in the present study.

## 5. Conclusions

This study suggests that the essential oils isolated from the leaves of *E. microtheca*, *E. procera*, *E. spatulata*, and *E. torquata* may be promising grain protectants against the lesser grain borer *R. dominica*. The essential oils have significant fumigant toxicity against insect pests which was augmented by increasing the concentration of essential oils and the exposure time. The *E. procera* essential oil, with a higher percentage of terpenes, was more toxic than others. Along with acute fumigant toxicity, *Eucalyptus* essential oils showed sublethal biochemical disturbances, including a reduction in protein, glycogen, and lipid contents, inhibition of digestive enzyme activity, and anti-nutritional effects on treated insects. These essential oils, as available natural agents, can be easily steam distilled from leaves of *Eucalyptus* trees, which are cultivated in many parts of Iran and several other countries. According to present and the previous studies, *Eucalyptus* essential oils are a complex mixture of several terpenic and non-terpenic constituents with multiple modes of action, and the chance of insect pests developing resistance is low. However, the resistance mechanisms of treated insect pests should be carefully evaluated. Further research is recommended to evaluate their toxicity on other insect pests and the side effects on stored products since they have a strong smell. Furthermore, although essential oils are safer on non-target organisms than conventional insecticides, performing more toxicological studies will be the next stage for their commercial application.

## Figures and Tables

**Table 1:** Chemical composition (%) of essential oils isolated from leaves of *Eucalyptus microtheca*, *E. procera*, *E. spatulata*, and *E. torquata*.

RI_calc_	RI_db_	Compound ^a^	*E. microtheca*	*E. procera*	*E. spathulata*	*E. torquata*
933	932	α-Pinene	5.6	14.0	3.9	20.0
954	954	Camphene	-	0.6	0.4	0.6
980	979	β-Pinene	5.1	0.5	1.3	0.8
1007	990	Myrcene	-	-	0.6	-
1025	1024	*p*-Cymene	0.9	1.3	3.8	2.6
1025	1029	Limonene	1.6	-	-	-
1032	1031	1,8-Cineole	12.1	21.3	27.1	24.2
1058	1059	γ-Terpinene	0.2	0.4	-	0.6
1062	1062	Artemisia ketone	-	-	0.5	-
1093	1086	Fenchone	0.3	-	-	-
1098	1096	Linalool	-	-	0.8	-
1098	1099	α-Pinene oxide	-	-	-	0.2
1101	1103	Isoamyl isovalerate	0.5	0.2	0.8	0.5
1104	1104	2-Methylbutyl isovalerate	-	-	-	0.2
1107	1114	*endo*-Fenchol	0.4	0.1	0.9	0.9
1115	1121	*exo*-Fenchol	1.4	1.1	-	-
1124	1128	*allo*-Ocimene	-	0.3	-	0.6
1130	1135	*trans*-Pinocarveol	1.2	5.0	2.3	0.2
1137	1140	Nopinone	0.4	-	-	-
1143	1144	*trans*-Verbenol	-	-	-	0.1
1143	1142	*trans*-Sabinol	3.8	14.6	7.6	2.0
1150	1149	Camphene hydrate	0.7	-	-	0.1
1153	1152	*iso*-Menthone	-	-	0.5	0.1
1157	1160	*iso*-Borneol	0.5	0.4	-	0.2
1166	1164	Pinocarvone	1.4	4.4	2.7	-
1169	1169	Borneol	1.5	0.9	1.4	1.8
1169	1177	Terpinen-4-ol	2.2	-	0.8	1.5
1178	1174	*iso*-Pinocamphone	-	0.4	-	-
1187	1185	Cryptone	-	-	-	0.3
1189	-	Unidentified ^b^	1.0	-	0.8	-
1190	1189	*trans-p*-Mentha-1(7),8-dien-2-ol	-	1.5	1.8	-
1192	1188	α-Terpineol	3.7	0.4	0.7	2.5
1199	1195	Myrtenal	1.2	-	-	-
1200	1195	Myrtenol	1.1	1.0	0.8	0.2
1202	1195 ^N^	*cis-iso*-Piperitenol	-	0.1	-	-
1212	1205	Verbenone	0.2	0.1	-	0.1
1220	1216	*trans*-Carveol	0.6	0.5	0.5	0.2
1230	1230	*cis-p*-Mentha-1(7),8-dien-2-ol	0.9	0.9	1.8	0.3
1232	1238	(*E*)-Ocimenone	-	0.2	-	0.1
1242	1241	Cuminaldehyde	1.3	-	2.5	0.4
1245	1243	Carvone	0.4	0.2	0.7	0.1
1249	1247	Carvotanacetone	-	t	-	0.1
1255	1252	Piperitone	0.3	0.1	0.9	0.3
1278	1275	Phellandral	-	-	-	0.1
1286	1284	(*E*)-Anethole	0.8	0.2	1.6	0.3
1288	1290	Thymol	0.4	0.1	1.1	0.1
1297	1298	*trans*-Pinocarvyl acetate	-	0.2	-	-
1300	1299	Carvacrol	0.5	t	0.6	0.1
1339	1337	2-Hydroxycineole acetate	0.6	t	0.4	-
1346	1346	α-Terpinyl acetate	-	t	0.2	-
1372	1374	*iso*-Ledene	0.3	t	-	0.3
1373	1374	α-Copaene	0.3	t	-	0.1
1390	1390	*trans*-β-Elemene	-	t	-	-
1410	1413 ^N^	β-Maaliene	-	0.1	-	-
1412	1409	α-Gurjunene	-	-	-	1.3
1420	1419	(*E*)-β-Caryophyllene	-	t	-	-
1429	1427 ^N^	γ-Maaliene	-	0.2	-	0.2
1434	1433	β-Gurjunene (=Calarene)	-	0.5	-	0.7
1442	1441	Aromadendrene	11.7	6.1	3.6	7.8
1459	1455 ^N^	Valerena-4,7(11)-diene	-	-	-	0.2
1463	1460	*allo*-Aromadendrene	2.6	1.3	1.0	1.8
1474	1477	γ-Gurjunene	0.4	0.1	-	0.2
1477	1479	γ-Muurolene	0.5	0.1	-	0.2
1487	1490 ^N^	Phenethyl isovalerate	-	-	0.5	-
1489	1490	β-Selinene	1.0	0.4	0.2	0.6
1496	1496	Viridiflorene	-	0.3	-	1.4
1499	1498	α-Selinene	0.5	-	-	-
1501	1500	α-Muurolene	0.3	-	-	0.1
1513	1513	γ-Cadinene	1.2	0.1	-	0.3
1520	1522	*trans*-Calamenene	0.6	0.1	-	-
1523	1523	δ-Cadinene	-	-	-	0.3
1562	-	Unidentified ^c^	3.0	2.1	2.8	2.7
1569	1567	Maaliol	2.8	1.8	2.5	1.4
1573	1584 ^N^	Boronia butenal	-	-	0.9	-
1574	1580 ^N^	*epi*-Globulol	-	-	-	0.3
1576	-	Unidentified ^d^	1.0	0.7	-	-
1582	1578	Spathulenol	4.6	-	-	0.7
1592	1590	Globulol	5.0	7.2	11.3	8.4
1598	1595	Cubeban-11-ol	2.3	2.0	2.9	2.4
1605	1600	Rosifoliol	1.0	0.3	0.3	1.0
1607	1602	Ledol	0.4	0.4	0.5	0.3
1618	-	Unidentified ^e^	1.3	0.7	1.0	0.5
1625	1629 ^S^	Rosifoliol isomer	1.2	0.5	0.6	1.6
1628	1628	1-*epi*-Cubenol	-	-	0.3	0.2
1629	1631	Muurola-4,10(14)-dien-1β-ol	1.1	0.3	-	-
1640	1640	τ-Cadinol	-	-	-	0.5
1642	1642	τ-Muurolol	1.1	0.1	-	-
1650	1650	β-Eudesmol	-	t	-	0.2
1655	1654	α-Cadinol	1.1	-	-	0.7
1656	1658	*neo*-Intermedeol	-	0.3	0.4	-
1659	1666	14-Hydroxy-9-*epi*-(*Z*)-caryophyllene	-	0.1	-	-
1674	1675	Cadalene	0.3	0.1	-	-
1900	1900	Nonadecane	0.4	-	-	-
		Monoterpene hydrocarbons	13.4	17.1	10.0	25.2
		Oxygenated monoterpenoids	36.6	53.6	56.6	36.0
		Sesquiterpene hydrocarbons	19.6	9.4	4.8	15.6
		Oxygenated sesquiterpenoids	20.5	13.0	18.9	17.6
		Others	2.2	0.4	3.8	1.2
		Total identified	92.3	93.5	94.0	95.6

RI_calc_ = Retention index calculated with respect to a homologous series of *n*-alkanes on a HP-5ms column. RI_db_ = Retention index from the Adams database [27] unless otherwise indicated: ^N^ NIST [28] and ^S^ Satyal [29]. t = trace (<0.05%). ^a^ Compound identification was based on MS fragmentation and RI comparison and are considered to be tentative. ^b^ MS (EI): 138 (4%), 121 (8%), 119 (12%), 108 (36%), 107 (22%), 105 (14%) 96 (22%), 95 (20%), 93 (16%), 91 (46%), 79 (100%), 77 (24%), 67 (14%), 65 (7%), 53 (7%), 41 (8%), 43 (7%). ^c^ MS (EI): 222 (10%), 204 (45%), 189 (47%), 161 (100%), 133 (34%), 121 (53%), 119 (53%), 109 (100%), 105 (64%), 95 (52%), 93 (63%), 82 (84%), 69 (59%), 43 (57%). ^d^ MS (EI): 222 (5%), 105 (41%), 204 (26%), 189 (36%), 163 (66%), 161 (58%), 149 (37%), 147 (37%), 133 (31%), 121 (44%), 119 (50%), 109 (65%), 107 (100%), 105 (65%), 93 (65%), 91 (58%), 81 (47%), 79 (46%), 69 (39%), 67 (35%), 59 (24%), 55 (21%), 43 (35%), 41 (21%). ^e^ MS (EI): 218 (15%), 203 (30%), 175 (28%), 161 (44%), 147 (36%), 133 (39%), 121 (47%), 120 (58%), 119 (57%), 107 (61%), 105 (79%), 93 (100%), 91 (89%), 79 (69%), 77 (54%), 67 (27%), 55 (36%), 41 (26%), and 43 (19%).

**Table 2:** The results of the Kolmogorov–Smirnov test and analysis of variance of the data obtained from the fumigant toxicity of *E. microtheca*, *E. procera*, *E. spatulata*, and *E. torquata* essential oils against the adults of *Rhyzopertha dominica*.

Essential Oil	Kolmogorov–Smirnov Test		Analysis of Variance					
			Concentration		Time		Concentration × Time	
	Z	Significant (Two-Tailed)	F (df = 5, 36)	*p*-Value	F (df = 2, 36)	*p*-Value	F (df = 10, 36)	*p*-Value
*E. microtheca*	0.879	0.423	134.425	<0.0001 *	33.507	<0.0001 *	1.496	1.4558 ^NS^
*E. procera*	0.778	0.580	214.959	<0.0001 *	57.999	<0.0001 *	2.584	0.0179 *
*E. spatulata*	0.778	0.579	139.085	<0.0001 *	12.275	<0.0001 *	0.723	0.6976 ^NS^
*E. torquata*	0.834	0.489	54.029	<0.0001 *	3.543	0.0394 *	0.371	0.9511 ^NS^

* Significant at α = 0.05. ^NS^: Not-Significant at α = 0.05. The number of tested insects is 1260 in each essential oil.

**Table 3:** Probit analyses of data obtained from the fumigant toxicity of *Eucalyptus microtheca*, *E. procera*, *E. spatulata*, and *E. torquata* essential oils against the adults of *Rhyzopertha dominica*.

Essential Oil	Time (h)	LC_50_ with 95% Confidence Limits (µL/L of Air)	LC_90_ with 95% Confidence Limits (µL/L of Air)	Relative Potency ^a^	χ^2^ (df = 4)	Slope ± SE	Sig. ^b^	*r* ^2^
*E. microtheca*	24	25.261 (21.295–29.077)	138.276 (101.151–227.377)	1.717	0.234	1.736 ± 0.216	0.994	0.997
	48	19.947 (17.721–22.002)	53.783 (47.142–64.084)	2.174	7.203	2.975 ± 0.268	0.126	0.987
	72	18.995 (16.969–20.853)	46.714 (41.554–54.431)	2.283	4.381	3.279 ± 0.289	0.357	0.989
*E. procera*	24	22.208 (18.749–25.721)	133.564 (95.976–223.511)	1.953	0.809	1.645 ± 0.196	0.937	0.989
	48	16.733 (14.778–18.582)	49.745 (42.984–60.362)	2.592	7.123	2.708 ± 0.238	0.130	0.998
	72	15.455 (13.861–16.953)	37.778 (33.575–43.973)	2.806	5.397	3.302 ± 0.279	0.249	0.971
*E. spatulata*	24	43.372 (37.683–49.779)	229.298 (158.106–432.583)	1.000	3.148	1.772 ± 0.246	0.533	0.947
	48	34.785 (30.576–38.779)	127.102 (101.882–177.469)	1.247	3.214	2.277 ± 0.257	0.523	0.965
	72	33.321 (29.673–36.747)	102.915 (86.441–132.090)	1.302	4.773	2.617 ± 0.266	0.311	0.959
*E. torquata*	24	37.728 (32.758–43.340)	201.490 (139.363–375.024)	1.150	0.797	1.761 ± 0.239	0.939	0.986
	48	32.284 (28.643–35.902)	116.741 (93.432–162.569)	1.343	1.042	2.296 ± 0.249	0.903	0.988
	72	31.567 (28.175–34.905)	105.017 (86.103–140.042)	1.374	2.582	2.455 ± 0.253	0.630	0.973

^a^ Relative potency = 24 h-LC_50_ value of *E. spatulata* essential oil/another LC_50_ value. ^b^ Since the significance (sig.) level is greater than 0.05, no heterogeneity factor is used in the calculation of confidence limits. The number of tested insects is 420 each time.

**Table 4:** The effects of low-lethal exposure to *Eucalyptus* essential oils (LC_30_ concentration) on energy reserves (µg/adult) (Mean ± SE) of *Rhyzopertha dominica* adults.

Treatment	Protein Content	Glycogen Content	Lipid Content
Control	126.17 ± 2.19 ^a^	57.22 ± 5.37 ^a^	6.93 ± 0.97 ^a^
*E. microtheca*	109.17 ± 1.92 ^b^	39.72 ± 1.47 ^b^	4.60 ± 0.23 ^b^
*E. procera*	101.33 ± 2.03 ^c^	35.78 ± 0.96 ^b^	4.80 ± 0.31 ^b^
*E. spatulata*	107.16 ± 2.52 ^bc^	37.44 ± 1.01 ^b^	5.47 ± 0.40 ^ab^
*E. torquata*	106.00 ± 2.75 ^bc^	39.28 ± 1.48 ^b^	6.20 ± 0.12 ^ab^
ANOVA	*F* = 17.02; df = 4, 10; *p* = 0.0002	*F* = 10.82; df = 4, 10; *p* = 0.0012	*F* = 3.76; df = 4, 10; *p* = 0.0407

Mean values in a column followed by different lowercase letters are significantly different on the basis of ANOVA with the LSD test (*p* < 0.05).

**Table 5:** The effects of low-lethal exposure to *Eucalyptus* essential oils (LC_30_ concentration) on esterase enzyme activity (µmol/min/mg protein) (Mean ± SE) of *Rhyzopertha dominica* adults.

Treatment	α-Esterase Activity	β-Esterase Activity
Control	0.041 ± 0.001 ^c^	0.119 ± 0.005 ^c^
*E. microtheca*	0.097 ± 0.001 ^a^	0.133 ± 0.005 ^bc^
*E. procera*	0.095 ± 0.002 ^a^	0.142 ± 0.009 ^ab^
*E. spatulata*	0.092 ± 0.003 ^a^	0.156 ± 0.005 ^a^
*E. torquata*	0.085 ± 0.001 ^b^	0.155 ± 0.008 ^a^
ANOVA	*F* = 145.06; df = 4, 10; *p* < 0.0001	*F* = 5.53; df = 4, 10; *p* = 0.0130

Mean values in a column followed by different lowercase letters are significantly different on the basis of ANOVA with the LSD test (*p* < 0.05).

**Table 6:** The effects of low-lethal exposure to *Eucalyptus* essential oils (LC_30_ concentration) on amylolytic and proteolytic activity (Mean ± SE) of *Rhyzopertha dominica* adults.

Treatment	Amylolytic Activity (mU/mg)	Proteolytic Activity (U/mg)
Control	0.400 ± 0.026 ^a^	0.121 ± 0.014 ^a^
*E. microtheca*	0.180 ± 0.038 ^b^	0.037 ± 0.007 ^b^
*E. procera*	0.193 ± 0.041 ^b^	0.038 ± 0.010 ^b^
*E. spatulata*	0.177 ± 0.047 ^b^	0.042 ± 0.005 ^b^
*E. torquata*	0.243 ± 0.042 ^b^	0.051 ± 0.018 ^b^
ANOVA	*F* = 5.70; df = 4, 10; *p* = 0.0118	*F* = 9.19; df = 4, 10; *p* = 0.0022

Mean values in a column followed by different lowercase letters are significantly different on the basis of ANOVA with the LSD test (*p* < 0.05).

**Table 7:** The effects of low-lethal exposure to *Eucalyptus* essential oils (LC_30_ concentration) on nutritional indices (Mean ± SE) of *Rhyzopertha dominica* adults.

Treatment	CI	ECI (%)	RCR (mg/mg/Day)	RGR (mg/mg/Day)
Control	7.69 ± 0.82 ^a^	3.06 ± 0.18 ^a^	0.57 ± 0.06 ^a^	0.017 ± 0.001 ^a^
*E. microtheca*	3.40 ± 0.58 ^b^	2.93 ± 0.77 ^a^	0.24 ± 0.04 ^b^	0.006 ± 0.002 ^b^
*E. procera*	4.42 ± 0.92 ^b^	2.97 ± 0.52 ^a^	0.32 ± 0.06 ^b^	0.009 ± 0.002 ^b^
*E. spatulata*	4.26 ± 0.81 ^b^	1.82 ± 0.69 ^a^	0.30 ± 0.05 ^b^	0.004 ± 0.001 ^b^
*E. torquata*	5.58 ± 1.29 ^ab^	2.90 ± 0.78 ^a^	0.40 ± 0.09 ^ab^	0.008 ± 0.002 ^b^
ANOVA	*F* = 3.75; df = 4, 30; *p* = 0.0137	*F* = 0.67; df = 4, 30; *p* = 0.6210	*F* = 3.75; df = 4, 30; *p* = 0.0137	*F* = 9.36; df = 4, 30; *p* < 0.0001

Mean values in a column followed by different lowercase letters are significantly different on the basis of ANOVA with the LSD test (*p* < 0.05). CI: Consumption Index; ECI: Efficiency of Conversion of Ingested food; RCR: Relative Consumption Rate; RGR: Relative Growth Rate.

## Data Availability

The data that support the findings of this study are available upon reasonable request.
